# Femtosecond laser filament induced condensation and precipitation in a cloud chamber

**DOI:** 10.1038/srep25417

**Published:** 2016-05-05

**Authors:** Jingjing Ju, Jiansheng Liu, Hong Liang, Yu Chen, Haiyi Sun, Yonghong Liu, Jingwei Wang, Cheng Wang, Tiejun Wang, Ruxin Li, Zhizhan Xu, See Leang Chin

**Affiliations:** 1 State Key Laboratory of High Field Laser Physics, Shanghai Institute of Optics and Fine Mechanics, Chinese Academy of Sciences, No. 390, Qinghe Road, Jiading District, Shanghai 201800, China; 2 IFSA Collaborative Innovation Center, Shanghai Jiao Tong University, Shanghai 200240, China; 3 MOE Key Laboratory of Advanced Micro-structured Materials, Institute of Precision Optical Engineering, School of Physics Science and Engineering, Tongji University, Shanghai 200092, China; 4 Center for Optics, Photonics and Laser (COPL), Laval University, Quebec City, Qc G1V 0A6, Canada

## Abstract

A unified picture of femtosecond laser induced precipitation in a cloud chamber is proposed. Among the three principal consequences of filamentation from the point of view of thermodynamics, namely, generation of chemicals, shock waves and thermal air flow motion (due to convection), the last one turns out to be the principal cause. Much of the filament induced chemicals would stick onto the existing background CCN’s (Cloud Condensation Nuclei) through collision making the latter more active. Strong mixing of air having a large temperature gradient would result in supersaturation in which the background CCN’s would grow efficiently into water/ice/snow. This conclusion was supported by two independent experiments using pure heating or a fan to imitate the laser-induced thermal effect or the strong air flow motion, respectively. Without the assistance of any shock wave and chemical CCN’s arising from laser filament, condensation and precipitation occurred. Meanwhile we believe that latent heat release during condensation /precipitation would enhance the air flow for mixing.

Recently, water condensation and precipitation in cloud chambers were successfully induced by UV lasers^1^,^2^,^3^ and by femtosecond laser-filaments^4^,^5^,^6^,^7^,^8^,^9^,^10^,^11^,^12^,^13^,^14^. While UV laser induced condensation was nicely explained to be principally due to OH radicals and/or H_2_O_2_ created by the UV laser^1^,^2^,^3^, the condensation and precipitation processes due to femtosecond laser filament^8^,^9^,^10^,^11^ is still not well understood.

Up until now, it was accepted that the plasma filament could generate Cloud Condensation Nuclei (CCN) which later contributed to condensation and precipitation. Dedicated study found that chemical products of laser-filament caused by photo-oxidation reactions, such as HNO_3_ etc. were sufficient to dissolve in surrounding water droplets; they could become important CCN^15^,^16^. In our work, the generation of the by-product HNO_3_ was observed by measuring the acidity and the NO_3_^−^ concentration in the laser-induced snow^8^,^9^,^14^. Besides that, in our first observation of precipitation reported in ref. 8, the following mechanism was proposed. In the cold zone (T = −28 °C) of the cloud chamber, inside the ‘pre-existing’ super-saturated zone (RH = 126%) based upon calculation, filament induced CCN’s (ions and the binary reaction H_2_O-HNO_3_) were produced at a high repetition rate (1 kHz) in the observed turbulent air flow. Consequently, large size (μm scale or larger) droplets or ice/snow particles condensed/precipitated out at the end. Later, when a 1 kHz laser was fired through a sub-saturated zone in a cloud chamber^9^, vortices induced by laser filament was demonstrated to be the main process to sustain a super-saturated zone by mixing air having a large temperature gradient between the filament zone and the cold plate. This super-saturated state would activate both ions and chemical by-products of filaments into CCN; the latter would grow in size in the vortices and fall down as snow/ice/water. In the above two sets of experiments, maintaining super-saturation for the filament-induced CCN to grow was believed to be the key to induce precipitation^8^,^9^.

However, in our first work^8^, the so called pre-existing super-saturated environment (T = −28 °C, RH = 126%) was based upon an idealized calculation; idealized in the sense that the cloud chamber was assumed to be perfectly clean, free of any contamination. In fact, our condition was far from that ideal. Before the femtosecond laser was fired, with the illumination of a green laser beam, side Mie scattered images given in^8^ showed that there were already droplets in the μm scale or larger (cloud) floating in the cloud chamber. This indicates that condensation has already occurred with the pre-existing background CCN (dust, aerosols, etc). These CCN would consume the excess water vapor and cause the relative humidity (RH) to decrease from a supposedly (idealized) super-saturated regime to a saturated or nearly saturated regime^17^. When the femtosecond laser filament was generated in the cloud chamber, the internal environment was no more super-saturated as the calculation presumed. In our other experiment under sub-saturated condition^9^, there were also pre-existing cloud droplets similar to the first experiment. In both cases, such pre-existing droplets and aerosols would compete with the filament generated ions and molecules such as HNO_3_ for the capture of water molecules and grow in size. We would expect that the pre-existing CCN’s and water droplets would be the principal source for precipitation. In this work, we shall propose a unified picture that can explain all our observations of precipitation in a cloud chamber. New experiments without laser filaments will be described to justify the unified picture. We shall first look at the excitation and relaxation of the filament zone so as to understand its principal consequences.

## Excitation and Relaxation of a Filament in Air

### Excitation

We consider pure air. It is well-known that a femtosecond laser pulse propagating in air with a peak power higher than the critical power for self-focusing will overcome diffraction (and dispersion) and self-focus towards a point. Before it reaches a singularity, the intensity inside the self-focusing pulse is strong enough to multiphoton/tunnel ionize the molecules in its path instantaneously thus generating a plasma. The plasma will de-focus the self-focusing pulse thus stopping the self-focusing. Such an event will repeat itself resulting in a series of connected self-foci which we call filament^18^,^19^,^20^,^21^,^22^,^23^. Ionization is the principal excitation process in which energy is transferred from the laser into the medium (filament or plasma zone). Some free electrons will attach to oxygen molecules. Moreover, the laser pulse in the filament will undergo self-phase modulation in both the neutral molecular gas and in in the plasma resulting in a spectacular spectral broadening called supercontinuum^18^,^19^,^20^,^21^,^22^,^23^,^24^,^25^. The high intensity inside the filament will generate third and higher harmonics^18^,^19^,^20^,^21^,^22^,^23^,^24^,^25^,^26^,^27^. It will align the molecules in air through rotational Raman excitation resulting in rotational wave packets. These wave packets will undergo many cycles of rotational de-phasing and revival well into the picosecond regime^28^,^29^,^30^,^31^. Nonlinear instantaneous pumping of molecular and ionic excited states resulting in fluorescence with gain will also take place^32^,^33^,^34^,^35^,^36^,^37^,^38^. Molecular dissociation and chemical reaction will take place inside the filament zone^15^,^16^,^33^,^34^ through dissociation and collision in the picosecond and nanosecond time scale. The longest time scale in the above mentioned excitation processes is of the order of nanosecond.

### Relaxation

We are interested in the energy that is retained inside the medium (air) after relaxation; i.e. after the laser pulse has gone. During the relaxation process, energy is lost through different types of radiation including fluorescence, molecular fragmentation and chemical reaction. Some of the radiations such as high order harmonics would be re-absorbed by the air molecules. The following collisional processes would transfer the excitation energy into the kinetic energy of the air molecules eventually. These are electron-ion recombination in the ps/ns time scale, collisional relaxation of rotational wave packets (ns), collisional relaxation of non-radiative excited molecules or fragments or chemical products (ns), etc. A hot filament zone is thus formed in the nanosecond time scale (note that acoustic relaxation and thermal diffusion are in a time scale much longer than 10 ns^39^). Because of such short time scale as compared to the slow acoustic and thermal motion in the microsecond and millisecond regime, we could consider that the density of air inside the filament zone is almost unchanged after all the relaxations. This would mean that there is a ‘sudden’ heating of the filament zone.

In ref. 39, Point *et al*. have generated a filament in air by focusing a Ti-sapphire laser at 800nm/50fs/5mJ with an f/35 (f = 1m) lens. They estimated that the temperature at the center of the filament zone could reach 1400 K. A sudden expansion resulting in the radiation of a shock wave was measured within 200 ns. It left behind a hot low density zone. The pressure inside the filament zone went back to atmospheric pressure in a few microseconds^40^. The resultant hot low density zone expanded to a few times the original size of the filament zone in about 500 μs. Convection resulting from heat conduction and gas motion took place. This is the source of strong air motion when the laser’s repetition rate is high. In our case, it is 1 kHz; i.e. the pulse to pulse interval (1 ms) was faster than the filament’s relaxation time of 90 ms measured by Point *et al*.^40^.

### Consequence

A filament after relaxation would give rise to some small quantity of new chemicals such as nitric acid, etc., shock waves radiating out from the filament zone and air motion (convection).

Our ‘normal’ cloud chambers are not clean; there are a lot of background CCN and water droplets. Some of the filament induced new molecules, ions and electrons would collide and dissolve/stick to the background water/ice droplets rendering the latter even more active. All these droplets will compete with the remaining filament induced chemical products to attract water molecules for condensation and precipitation. In our experiments using the 1 kHz repetition rate laser pulses, some of these droplets would be shattered by strong field inside the filament zone. This would increase the number of existing background droplets and CCN’s for precipitation^6^. (From now on, background droplets or background CCN’s refers to those with and without filament induced chemicals sticking onto them.) We expect that the chance for the individual filament produced molecules to grow into large droplets would be minimal as compared to the growth of background droplets.

Meanwhile, the shock waves would compress the moist air in and surrounding the filament in a few hundred ns^40^. This compression would result in a smaller air volume while the water vapor content is almost unchanged. Consequently, the high density/pressure shock zone would become saturated or supersaturated. Because there are a lot of background CCN and water droplets, condensation would take place inside these shock zones which radiate out into the free space of the cloud chamber. Such condensation would eventually mix with the background and become part of the background CCN.

The remaining consequence of filamentation would be air motion that would last in the tens of ms time scale. It would be this air motion which would be the principal mechanism to induce precipitation.

## A Unified Picture of Precipitation

This unified picture is similar to the one used in explaining the precipitation induced by a filament in a sub-saturated zone^9^,^11^ with the exception that in the background, there were already a lot of background and shock-induced CCN’s and water/ice droplets. Thus, our unified picture runs as follows.

**Figure 1:**
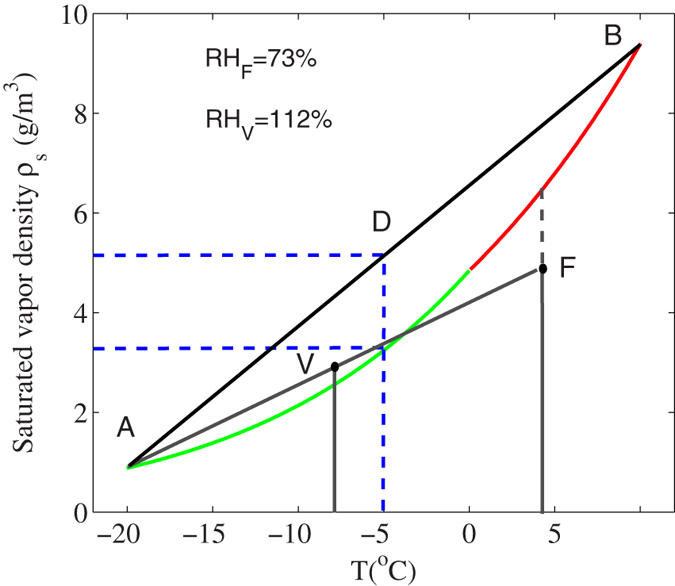
Saturation water vapor density (relative to ice in green and relative to water in red) vs temperature illustrating the idea of linear homogeneous mixing of air between two saturated regions (A,B) resulting in a mean temperature (D) where super-saturation occurs. Linear homogeneous mixing of air near the filament (F) and cold plate (A) mentioned in^11^ are also reconsidered with point V referring to the saturated vapor density of mixed air.

High repetition filamentation would sustain a quasi-continuous thermal air flow (convection) after filament relaxation inside the cloud chamber. The air flow, be it vortices or turbulence or both^12^,^13^ will mix up the moist air in a zone where there is a large temperature gradient. According to the empirical formula of saturated water vapor pressure P_ice_ (relative to ice) or P_liq_ (relative to liquid water) versus temperature T^41^,^42^,


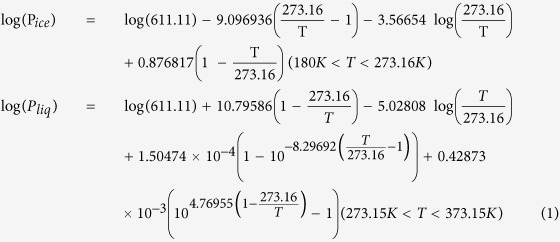


mixing air across a large temperature gradient would result in supersaturation at the average (mean) temperature. For example, in Fig. 1, the curve represents the water vapor saturated density ρ_s_ relative to ice (below 0 °C, green curve) and relative to liquid water (above 0 °C, red curve) (ρ_s_ = P_ice/liq_M/RT, where M is the molar mass of water and R the perfect gas constant) as a function of temperature (based upon Equation 1) in a cloud chamber. If the air between two saturated zones, A and B, having a temperature gradient between −20 °C and 10 °C were mixed linearly and thoroughly, the mean temperature in the mixed region would become −5 °C. That is to say, the linear mixing follows the straight line between points A and B. At this average temperature of −5 °C, the region becomes supersaturated (point D). The existing background CCN’s and water/ice droplets would start to condense out and precipitate in the super saturated state under quasi-continuous mixing.

This mixing (air flow) could be enhanced by the release of latent heat during condensation/precipitation. In our previous analysis of thermodynamic air flow created by filamentation in air^12^, it was found that the theoretical maximum velocity of updraft, which did not take into account the release of latent heat, was consistently smaller than the experimental one. We now believe that this discrepancy could have been due to latent heat; i.e. latent heat might have played a role in our experiments to enhance the air flow rendering the mixing process even more efficient. In the following discussion, it will be assumed that latent heat is part of the driving force of air flow (mixing) and will not be mentioned anymore.

## Explanation of Previous Experiments

**Figure 2:**
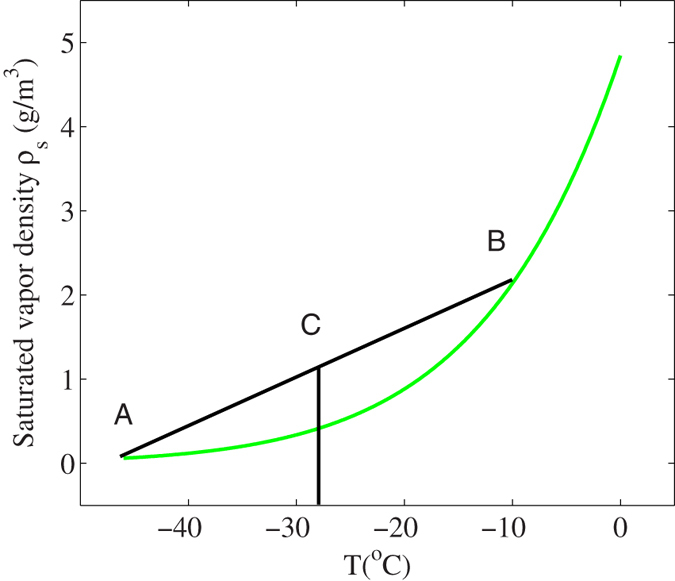
Saturated vapor density ρ_s_ relative to ice Vs. Temperature (T) from −40 °C to 0 °C (green curve). The straight line represents linear mixing of air between points A and B. The mean temperature is at point C where there is supersaturation.

We can now explain consistently our previous experiments. In the first experimental observation of precipitation^8^, the strong (multiple) filaments induced a violent turbulence which mixed a large zone (~8 cm) above the cold plate. As discussed in the introduction, before filamentation, there was a lot of pre-existing background aerosols and CCN’s inside the cloud chamber. Since the temperature inside the interaction zone was very low, between −10 °C and −46 °C, the background CCN’s would consist of Ice Nuclei (IN) together with super cooled water droplets. These CCN’s would consume the background water vapor in the otherwise idealized super-saturated environment. The consequence was that there was no more super-saturation inside the cloud chamber before filamentation. The best was saturation or near (sub-) saturation^43^. Filaments were generated after cooling down the cloud chamber for about 30 min. If we assume saturation, the consequence can be seen in Fig. 2. The mean temperature between region A (−46 °C) and B (−10 °C) is at point C (−28 °C) where super-saturation occurs. The consistent quasi-continuous turbulence induced by the relaxation of the high repetition filaments would maintain this super-saturated state. Hence, further condensation of the background CCN’s followed by precipitation would take place giving rise to snow.

Our second experiment on the generation of precipitation in a sub-saturated zone^9^,^11^ was already explained in a similar way. It was due to air mixing by the filament induced vortices except that in the present explanation, it was the pre-existing background CCN’s in the cloud chamber that would grow in size in competition with the filament induced chemicals. The background CCN’s would consist of water droplets as well as IN from the zone near the cold plate (−20 °C), apart from the existing aerosols. Besides that, since the filament was generated in a zone at a temperature of 4.5 °C and the cold plate was at around −20 °C, we need to introduce the saturation curves with respect to both liquid water (T > 0 °C) and ice (T < 0 °C). This is shown in Fig. 1. By mixing air around the filament (T = 4.5 °C RH = 73% point F in Fig. 1) and near the cold plate (T = −20 °C, RH = 100% point A in Fig. 1), the vortices (air mixing) would keep a relative humidity of 112% at their average temperature of ~−12 °C (point V in Fig. 1) (which was given as 105% in^11^ with saturation ratio relative to water only). This value is high enough for the growth of the background CCN’s to condense and precipitate.

## Experimental Support of the Idea of Thermal Air Flow

If thermal effect and the subsequent air flow (turbulence and vortices) caused by the filaments were the core reasons of inducing precipitation, we should expect to see condensation and precipitation if we use a heating source only or blowing air only inside the cloud chamber. To justify this idea, two independent experiments were carried out inside the same cloud chamber used in our first precipitation experiment^8^. One was to use a heating pipe to imitate the filament heating and the other was to use a fan to imitate air flow induced by the filament. In these two experiments, no chemical was created and no shock wave was generated.

## Experiment 1: pure heating

**Figure 3:**
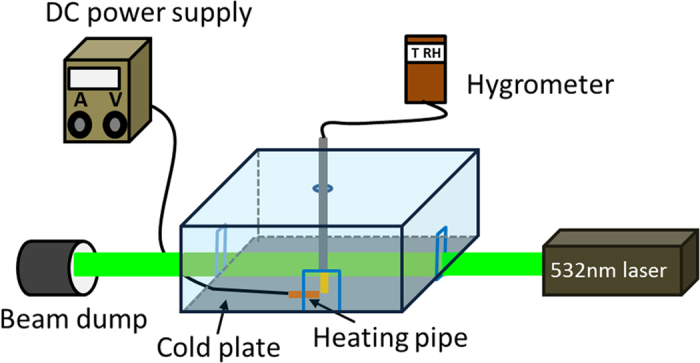
The heating pipe experiment setup.

A 6 mm x 20 mm electric heating pipe was introduced inside the chamber (Fig. 3). A DC power supply was used to control the heating power. At the same time, a hygrometer was put inside the chamber near the pipe to monitoring the humidity and temperature change. A 532 nm laser beam with an expanded diameter of 2 cm was used as the probe beam (Fig. 3). The probe laser beam was set in the middle of the chamber which was 2.0 cm away from the axis of the heating pipe and hygrometer. It was to avoid the strong scattered lights by the pipe and hygrometer. Both the hygrometer and heating pipe were set above the cold plate at a vertical distance of 1.0 cm. This position was the same as the position of the filament in our previous experiment.

**Figure 4:**
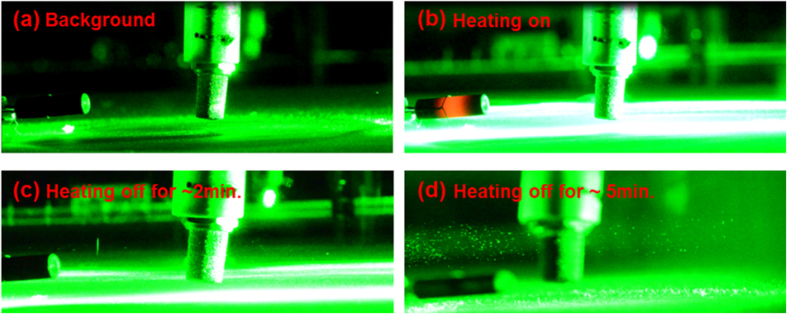
Side scattered images of scene inside chamber when a heating pipe was used.

The cloud chamber was kept cold (at −46 °C at the bottom) in the first 30 min. while the water bath at the top of the chamber was kept at 23 °C. Then, heating was turned on during the next 15 min, and switched off for the last 15 min with the cooling working all the time. The scene inside the chamber was recorded by a digital camera (Nikon 7100) from the side (See supplementary material). It was found that when the heating was turned on, it ‘cleaned’ the air nearby at the first few seconds because of the evaporation of water droplets under the illumination of the infrared rays from the heating element. (This infrared radiation from the heating element also heated up a large area right below the heating element so that there was no accumulation of snow over there.) But soon a layer of mist near the cold plate moved in and grew thicker and thicker. The strong scattered light from the thick fog saturated the video images (see both supplementary material and Fig. 4b). The thermometer showed that the heating had caused the temperature of air near the hygrometer to increase by ΔT >10 °C. When the heating was switched off, it was found that in ~2 min., a few large size (presumably) ice particles were seen dropping down to the cold plate quickly leaving a ‘long’ trail on the image (e.g. Fig. 4c with exposure time ~1/50 s). And in ~5 min. when we scanned the chamber from the bottom to the top by the green probe beam, we saw that the whole cloud chamber was filled with plenty of (‘presumably’) ice particles (Fig. 4d), some of which were floating with the air flow motion and others heavy enough dropping down to the cold plate directly. Snow covering the whole cold plate was collected and weighed at the end. With the heating pipe introduced, the snow weight was ~3.13 ± 0.7 g compared to ~4.3 ± 0.58 g without introducing the heating pipe. The total weight of snow in the thermal case had decreased. This might be ascribed to the evaporation of snow caused by the strong infrared radiation from the heating element as explained before.

**Figure 5:**
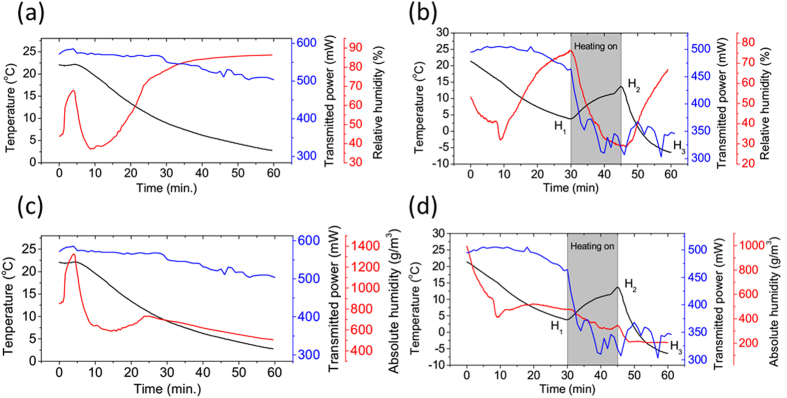
(**a,c**) Background relative humidity (**a**) or absolute humidity (**c**), temperature and transmitted power vs time; (**b,d**) Relative Humidity (**b**) or absolute humidity (**d**), temperature and transmitted power vs time when a heating pipe was used.

The temporal evolution of temperature, relative humidity (RH) and transmitted power of the probe laser were measured while the cooling of the cold plate was working all the time. In order to assessing the evaporation and condensation process intuitively, the absolute humidity (AH) was calculated according to *AH* = *RH* × *P*_*s*_*M/(RT)*, where *P*_*s*_ is the saturated water vapor pressure at absolute temperature *T*, *M* , the molar mass of water, *R*, the molar gas constant (Fig. 5c,d). Figure 5a shows that before heating, the temperature decreased gradually with time. The transmitted probe laser power did not change too much, which was near ~500 mW at the end. The relative humidity reached a plateau at around 85% (Fig. 5b). As the temperature decreased, the saturation vapor pressure should decrease exponentially (Fig. 1). The excess water vapor condensed out as droplets, so the AH decreased as shown in Fig. 5c. However, when the heating pipe was turned on (Fig. 5b), a sharp increase of the temperature near the hygrometer from ~3.5 °C to ~13.8 °C (H_1_ to H_2_) was observed. This was accompanied by a sharp decrease of both humidity and transmitted probe laser power. As shown in the supplementary materials, water condensation appeared quickly near the cold plate during this period. It scattered the probe laser strongly in all directions in a short time resulting in a sharp decrease of the transmitted power (from ~500 mW to ~300 mW). During this period, infrared radiation from the hot pipe heated up the moist air and evaporated the water droplets around into vapor. This should increase the corresponding AH, since there were now plenty of water vapor around and the saturation vapor pressure should exponentially increase also. But in fact (Fig. 5b,d) from H_1_ to H_2_ the AH decreased. This indicated that water vapor which condensed out as droplets was much more than that evaporated from droplets. This agreed well with what is shown in the supplementary video.

When the heating was switched off after heating for 15 min., (H_2_ → H_3_), infrared radiation stopped and the temperature decreased sharply. The temperature decrease caused a decrease of saturation vapor pressure exponentially. Water vapor condensed out as droplets and the AH decreased sharply also in the first ~2 min. However, later, AH became stable (Fig. 5d the red curve from H_2_ → H_3_). This indicated that the amount of pure water vapor in air did not change too much from then on even though the saturation vapor pressure decreased continuously. In the experiment, we also observed that precipitation occurred after the heating was switched off for ~2 min. (shown in the supplementary video). This coincidence indicated that precipitation occurred at that time through the collision-coalescence process between water droplets, and during this period not too much vapor was consumed. Also the transmitted power of the probe laser did not increase too much (fluctuating around 300mW). This might be because the scattering from the large size ice particles formed during this period attenuated the laser power.

**Figure 6:**
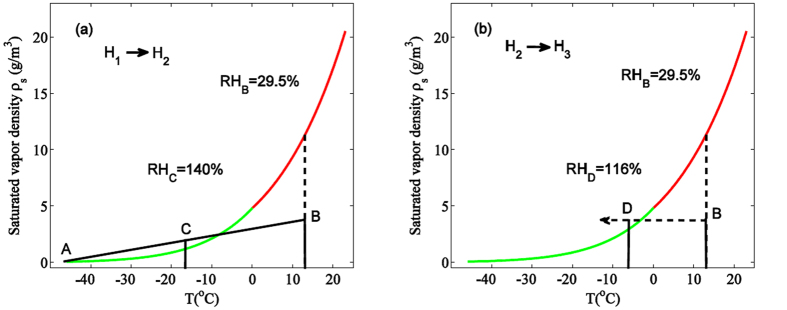
Mixing of air (**a**) when the heating was on and (**b**) when the heating was switched off. Saturated vapor density ρ_s_ relative to ice (from −46 °C to 0 °C) is in green and that relative to water (from 0 °C to 23 °C) is in red.

To explain the observed phenomenon, the saturated vapor density vs. temperature was calculated from −46 °C *to* 23 °C based upon Eq. 1. When the heating was on (from H_1_ to H_2_ in Fig. 5b), the heated air underwent convection through accelerated thermal air motion inside the chamber. During this heating cycle, when air was mixed between H_1_ (T = 13.8 °C RH = 29.5% point B in Fig. 6a) and the cold plate (point A in Fig. 6a), a super-saturated state of RH = 140% could be obtained (point C in Fig. 6a). When the heating was off, the evolution from H_2_ to H_3_ (Fig. 5b) during 15 min. would lead to the change in temperature and RH from point B (T = 13.8 °C RH = 29.5%, Fig. 6b) to point D (T = −6.5 °C, see Fig. 5b, black curve at H_3_) where RH~116% (relative to ice) was reached. Meanwhile not only the air near the hygrometer, but also the air in the whole cloud chamber suffered a sudden decrease of temperature since the cooling was still on at the bottom. This temperature decrease was ΔT > 20 °C. At the beginning, inside the chamber, the humid air was sub-saturated, but it became super-saturated soon across the whole chamber when the heating was off. The background was full of pre-existing CCN as discussed in the introduction. Under such a high super-saturated condition, these CCN consumed the water vapor around and condensed/precipitate out as water/ice droplets, which appeared as mist/fog near the cold plate or snow fall across the whole chamber.

## Experiment 2: pure air flow

**Figure 7:**
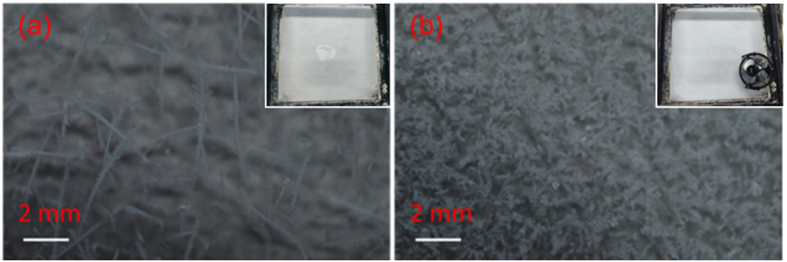
Close up shots of snow formation on the cold plate (**a**) background (**b**) with a fan blowing for 30 min. The cloud chamber was pre-cooled for 30 min. before the fan was turned on. The scene of snow formation on the whole cold plate without (**a**) and with fan (**b**) at the end was shown in the insets correspondingly.

**Figure 8:**
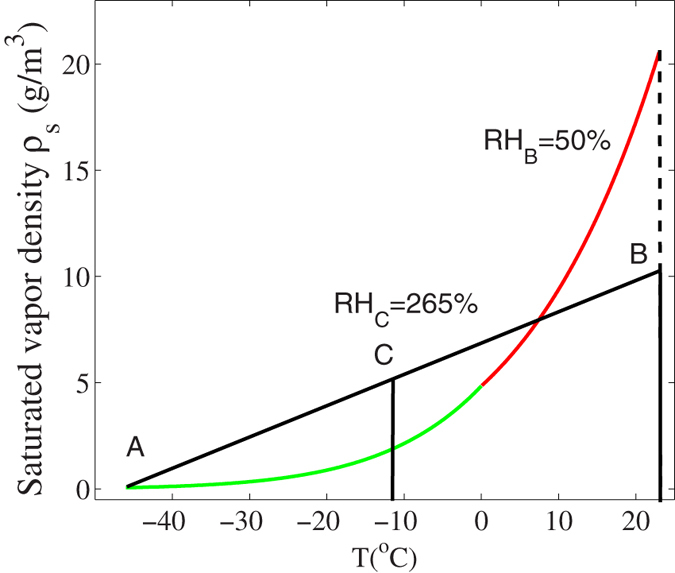
Calculated saturated vapor density ρ_s_ relative to ice (from −46 °C to 0 °C in green) and water (from 0 °C to 23 °C in red) Vs. temperature (T).

We also circulated air inside the cloud chamber. This was done by blowing air with a fan (with a diameter of ~10cm) sitting inside the cloud chamber. The total experimental duration was 60 min, with cooling in the first 30 min. and both cooling and circulating air in the last 30 min.. The strong air blown by the fan (with a speed of ~1 m/s, while the speed of airflow induced by the filaments was estimated to be 60 cm/s^8^) emphasized the effect of air mixing induced by the filaments. At the end, snow formed on the cold plate was much thicker than that of the background. It weighed ~7.61 ± 1.67 g, which was almost twice that in the case without blowing air (~4.3 ± 0.58 g). The shape and size of the snow/ice particles produced with and without the fan blowing were different. This could be seen by comparing Fig. 7a,b. We can also estimate the super-saturation ratio caused by the fan (Fig. 8). Mixing the air from the top of the chamber (point B, T = 23 °C; RH = 50%) with the air at the bottom (point A, T = −46 °C; RH = 100%) would lead to a very high supersaturated state at their averaged temperature (point C) ~RH = 265% resulting in condensation and precipitation efficiently.

## Discussion of the two non-filamentation experiments

In the pure heating experiment, the cloud chamber was kept at a temperature gradient of ~70 °C in a vertical height of 20cm, which was especially sharp near the cold plate^44^. The electric heating source inside the chamber introduced a thermal disturbance, which accelerated the air flow motion inside. Humid air near the heating pipe would follow the air flow motion and mix with the air near the cold plate. The mixed air got super-saturated (RH ~ 140%, Fig. 6a). With such a high super saturation ratio, the background CCN (IN) would quickly grow in size and become large size liquid/solid particles. The latter were observed through scattering of the green probe beam in the form of fog or mist. After the heating was turned off, the cooling brought the chamber back to a super-saturated state contributing again to condensation and precipitation (Fig. 6b).

In the experiment of purely blowing air, the air inside the whole cloud chamber was mixed resulting again in a super-saturated state with the RH reaching 265% (Fig. 8). The background CCN (IN) would quickly grow in size and become large size liquid/solid particles.

These two experiments demonstrated that, without the generation of any chemical CCN or shock waves, only a heating source or a blowing fan could trigger the water condensation/precipitation processes in the diffusion cloud chamber. These results indicated that there were plenty of impurities in the cloud chamber ready to become CCN as long as the super-saturated state was satisfied. This would mean that chemical products from the filaments were not the main CCN for inducing the condensation and precipitation process. Shock wave induced condensation would not play an important role either. Shock induced condensation might have merged into the background CCN. In filament induced precipitation, the formation of a supersaturated state induced and sustained by the filament induced air mixing had played the key role.

## Conclusions

A unified picture that could explain filament induced precipitation in a normal diffusion cloud chamber has emerged. Among the three major consequences of filamentation inside the cloud chamber, namely, the generation of shock waves, the creation of a small quantity of chemicals and the generation of strong thermal air flow, the last one turns out to be the principal reason that is responsible for inducing precipitation. In such a normal cloud chamber having already a lot of background CCN (IN), the strong air flow left behind by the filament would mix the moist air having a large temperature gradient into a super-saturated state. The background CCN (IN)’s, including those adhered by the filament generated chemicals through collision, would quickly grow in size and precipitate into water/snow/ice. This conclusion was supported by further experiments using either a pure heating source or a pure blowing fan inside the cloud chamber to simulate filament induced air flow without shock waves or without newly created chemicals. In both cases, condensation and precipitation occurred. Meanwhile our current understanding indicates that the latent heat released during condensation/precipitation would play the role of enhancing the air flow rendering mixing more efficient.

Before ending, we like to underline that despite the importance of air flow, the filament generated chemicals such as HNO_3_ would still be left behind in the precipitation. ‘Acid rain’ would be an eventual concern if the experiment were to be carried out in the atmosphere. However, a very recent experimental finding by the Geneva group^45^ demonstrated that naturally existing NH_3_ in the atmosphere would neutralize a major part of the filament generated HNO_3_ resulting in the creation of NH_4_NO_3_.

## Methods

The experiments were carried out in a laboratory diffusion cloud chamber of 0.5m × 0.5 × 0.2 m^3^ which was filled with ambient air. A vertical temperature gradient was maintained in the chamber by using a refrigerating machine to cool the bottom base plate at a temperature as low as −46 °C, while the top of the chamber was kept natural cooling. Water vapor diffuses towards the bottom base plate from a reservoir in 45 cm × 45 cm square frame with a cross-section of 5 cm × 2 cm downward-pointing triangle mounted at a height of 17 cm relative to the bottom inside the chamber. The quantity of water vapor can be controlled by adjusting the electric current of a heating wire submerged in the water at different values. The heating electric current of 0.5 A was applied in the present experiments. The relative humidity at the top of the cloud chamber and the typical vertical temperature distribution in the cloud chamber were measured with a ZDR-F20 Humidity Logger and a thermistor thermometer, respectively.

The heating pipe used was a DC resistance heating tube of 20 mm (length) × 6 mm (diameter) and the small fan used was a normal 5 W AC mini fan as described in the experimental part. A probe beam, which was delivered from a continuous semiconductor laser with 2.0 W output power and a wavelength of 532 nm, (with a diameter of 2.0 cm) propagated horizontally to the cold plate in order to illuminate the interaction zone. The side Mie scattering was recorded by a digital single-lens reflex camera (Nikon D7000, 3264 × 4928 pixels) with a macro lens (AF 60 mm/2.8D). Water condensation, ice particle generation, as well as air flow motion were observed by naked eye and recorded by the digital camera.

## Additional Information

**How to cite this article**: Ju, J. *et al*. Femtosecond laser filament induced condensation and precipitation in a cloud chamber. *Sci. Rep*. **6**, 25417; doi: 10.1038/srep25417 (2016).

## Supplementary Material

Supplementary Information

Supplementary Video S1
