# Supplementary material for: Femtosecond laser filament induced condensation and precipitation in a cloud chamber
Source: Sci Rep. 2016 May 5;6:25417. doi: 10.1038/srep25417 (PMC4855206; doi:10.1038/srep25417)
Supplement: Supplementary Information [file srep25417-s1.pdf]

Femtosecond laser filament induced condensation and precipitation in a cloud chamber

Jingjing Ju[1], Jiansheng Liu[1], Hong Liang[1], Yu Chen[1], Haiyi Sun[1], Yonghong Liu[1,2], Jingwei Wang[1], Cheng Wang[1], Tiejun Wang[1], Ruxin Li[1], Zhizhan Xu[1], See Leang Chin[3]

[1]State key lab of high laser field physics, Shanghai Institute of Optics and Fine mechanics, Chinese Academy of Science, No. 390, Qinghe Road, Jiading District, Shanghai 201800, China

[2]MOE Key Laboratory of Advanced Micro-structured Materials, Institute of Precision Optical Engineering, School of Physics Science and Engineering, Tongji University, Shanghai 200092, China

[3]Center for Optics, Photonics and Laser (COPL), Laval University, Quebec City, Qc G1V 0A6, Canada

Corresponding authors:
zzxu@mail.shcnc.ac.cn; slchin@phy.ulaval.ca

Supplementary video information

Video 1：Condensation and precipitation induced by a heating pipe in a cloud chamber
